# P-1886. Biopreparedness Education in US Medical Schools

**DOI:** 10.1093/ofid/ofaf695.2055

**Published:** 2026-01-11

**Authors:** Anna Kilzer, James Lawler, Angela Hewlett

**Affiliations:** University of Nebraska Medical Center, Omaha, NE; University of Nebraska Medical Center, Omaha, NE; University of Nebraska Medical Center, Omaha, NE

## Abstract

**Background:**

The 2014-2016 Ebola outbreak and the COVID-19 pandemic highlight the risks that infectious diseases present to public health. A key component of biopreparedness is training the next generation of physicians to manage patients with high consequence pathogens. The University of Nebraska Medical Center, a Regional Emerging Special Pathogen Treatment Center (RESPTC), offers a biopreparedness and biocontainment Enhanced Medical Education Track (EMET) for medical students. Due to the lack of data on other formal medical student training programs in biopreparedness, we conducted a national survey to assess similar training and education programs at other US medical schools.

**Methods:**

A 9-question survey was sent via email or mail to medical school curriculum leaders at the 158 Liaison Committee on Medical Education (LCME) accredited medical education programs in the US. The survey assessed available opportunities in biopreparedness and biocontainment education and gauged interest in educational programming in these areas.

**Results:**

Thirty-one total survey responses were received. Twenty-three responders (74%) reported no formal biopreparedness or biocontainment curriculum at their institution. When asked to evaluate medical student readiness to respond to public health emergencies involving special pathogens, 74% of respondents chose “average”, “below average”, or “poor”. Regarding potential integration of educational resources into their curriculum, most respondents selected “online curriculum (videos, interactive programs, didactics) that can be viewed at your institution” and “online course on biopreparedness and biocontainment hosted by a RESPTC”.

**Conclusion:**

The vast majority of US medical schools responding to our survey do not offer formal training in biopreparedness or biocontainment. Additionally, many medical school curriculum leaders believe there is opportunity for improvement in student preparedness for public health emergencies involving special pathogens, and they prefer future trainings to be developed in an online format. These results demonstrate the paucity of educational options in biopreparedness in US medical schools and emphasize the urgent need for development of educational content in this area.

**Disclosures:**

James Lawler, MD, MPH, FIDSA, Arkansas Hospital Association: Honoraria|Seqirus-CSL: Advisor/Consultant Angela Hewlett, MD, MS, Forecast Orthopedics: Advisor/Consultant|Mapp Biopharmaceutical Inc.: Grant/Research Support

